# A framework of connections between soil and people can help improve sustainability of the food system and soil functions

**DOI:** 10.1007/s13280-017-0965-z

**Published:** 2017-11-24

**Authors:** Bruce C. Ball, Paul R. Hargreaves, Christine A. Watson

**Affiliations:** 1 Crop and Soil Research Group, SRUC, West Mains Road, Edinburgh, EH9 3JG UK; 2 Future Farming Systems Group, SRUC, West Mains Road, Edinburgh, EH9 3JG UK; 3 Crop and Soil Research Group, SRUC, Craibstone Estate, Aberdeen, AB2 9YA UK

**Keywords:** Agroecology, Diversity, Integration, Soil quality

## Abstract

Globally soil quality and food security continue to decrease indicating that agriculture and the food system need to adapt. Improving connection to the soil by knowledge exchange can help achieve this. We propose a framework of three types of connections that allow the targeting of appropriate messages to different groups of people. Direct connection by, for example, handling soil develops soil awareness for management that can be fostered by farmers joining groups on soil-focused farming such as organic farming or no-till. Indirect connections between soil, food and ecosystem services can inform food choices and environmental awareness in the public and can be promoted by, for example, gardening, education and art. Temporal connection revealed from past usage of soil helps to bring awareness to policy workers of the need for the long-term preservation of soil quality for environmental conservation. The understanding of indirect and temporal connections can be helped by comparing them with the operations of the networks of soil organisms and porosity that sustain soil fertility and soil functions.

## Introduction

The perceived need to maintain and increase agricultural productivity to sustain economic growth encourages pressure on the soil, causing soil degradation. This situation is serious with a recent report by FAO and ITPS (2015) concluding that the majority of the World’s soil resources are at best only in fair condition, with 33% of land moderately to highly degraded. Soil degradation and the consequent loss of productive soils by erosion (Labrière et al. 2015) have been recognised as having a substantial impact on food security by reducing crop productivity (Lal 2009a; Bindraban et al. 2012) and the nutritional quality of food through nutrient mining leading to micronutrient deficiencies (Jones et al. 2013). Soil degradation has other effects on human health (Abrahams 2002; Oliver and Gregory 2015), such as heavy metal and PCB contamination (Filippelli and Laidlaw 2010; Cachada et al. 2012). Soil degradation also impairs important soil functions within landscapes. These include nutrient cycling, water retention, biodiversity and habitat, storing, filtering and transforming compounds and support through the provision of physical stability (Nannipieri et al. 2003; Bronick and Lal 2005; Tolon-Becerra et al. 2011); these are known collectively as ecosystem services (Lal 2010).

Stabilisation and reversal of soil degradation are vital to food security and ecosystem services; concepts that can be expressed as soil security. Soil security relates to the need for improved management of soil for the continued production of food, fibre and fresh water, along with the ability to contribute to energy and climate sustainability and to maintain the biodiversity of ecosystems (McBratney et al. 2014). Improved soil management and awareness of soil condition are vital for all agriculture but particularly for sustainable agricultural techniques such as organic farming and reduced tillage that depend heavily on recycling of inputs with significant emphasis on conserving soil and soil functions (Maeder et al. 2002; Watson et al. 2002; Gomiero et al. 2011). We call this soil-focused farming. Promotion of such systems is clearly relevant to farmers and stakeholders.

Agroecological systems such as organic farming and other forms of soil-conserving sustainable agriculture can compete with conventional agriculture and have the potential to maintain food productivity while improving health and diet as well as sustaining soils, waters and ecosystems (Halberg et al. 2015; IPES-Food 2016). Agroecological systems are two to four times more energy efficient than conventional agriculture (IPES-Food 2016). They are thus important for the future because of their reduced reliance on fossil fuels for cheap energy and fertilisers and on the naïve idea that technology can continue to solve our problems (Weis 2010). Agroecology, with such emphases on efficient input use and environmental benefits, is also compatible with ideas of sustainable intensification (Lampkin et al. 2015). Concepts of long-term sustainability of production and soil function are important for scientists to demonstrate and are vital goals for policy makers.

Experience in soil-focused farming and agroecology at SAC (the former Scottish Agricultural College) and SRUC (Scotland’s Rural College) was gained in separate long-term experiments on reduced tillage agriculture and organic farming systems in a moist, temperate climate. Both organic farming (Taylor et al. 2006) and long-term no-till (Soane and Ball 1998) proved sustainable over 20 years’ duration in terms of yield, soil quality and ecosystem services, perhaps as a result of soil-aware management.

Farmers and stakeholders need to be made aware of the importance of management for long-term sustainability of soil and food production, and we believe this could be facilitated by improving their connection with the soil. Also human society as a whole needs to become more aware of its connection to the soil and realise the dependence on soil for food, biomass and the functions it provides to maintain the biosphere (FAO and ITPS 2015). At a symposium on global soil security in 2015, improving connectivity was seen as important for increasing awareness and understanding of soil security both in the general public and in agricultural policy (Morgan et al. 2015).

SAC and SRUC have consistently promoted and delivered knowledge transfer and exchange on soils over many years to these three target audiences, i.e. farmers and stakeholders, policy makers and scientists and society as a whole. Knowledge transfer for farmers has focused on the connections between crop and ecosystem services through soil management (mainly tillage and organic farming) and soil quality, increasingly assessed using visual soil evaluation (REF 2014; AHDB 2016). We perceive visual soil evaluation as a direct connection to the soil with awareness increased simply by digging it up and looking at it. This can be formalised in visual soil evaluation by scoring soil condition from the appearance of the soil broken-up on a spade or on the side of a pit (Ball et al. 2017).

In our knowledge exchange discussions with researcher colleagues and policy makers, we have used long-term trends in soil quality under no-till and organic farming at SRUC to show their environmental benefits (Soane and Ball 1998; Taylor et al. 2006; Cloy et al. 2016) and the consequences of loss of long-held knowledge on land management (Scherbatskoy et al. 2015). We identify these as temporal connections of soil to land users and wider society through trends in soil quality, land management and climate change.

We have engaged with the public through talks and demonstrations such as soil-to-plate links via food and soil to global change links via greenhouse gas emissions. Further involvement has included artists and church groups in developing more ecological connections with the soil. These latter connections permit the development of ‘cognisance’ or a deep awareness linked to ecological consciousness that Grunwald et al (2017) considered can motivate appropriate actions within a range of contexts to ensure soils security. Such innovative approaches can improve the connection of people to soil by boosting the soil knowledge of land users and increasing the understanding of the consequences of food choices and soil functions by society in general, thereby improving the sustainability of the food system and increasing soil security. These connections that do not involve actual soil contact are perceived as indirect connections to the soil.

We consider that the distinction of these three types of connection, direct, indirect and temporal within a framework of soil connectivity is appropriate to facilitate delivery of different messages about soil to three distinct target audiences identified above (Table 1). Mechanisms of connection for each target audience are summarised in Table 2. Our objective is to develop these novel ideas of soil connectivity from our own research and experience and from examples in the literature and on-line. We then demonstrate how this framework can be used to improve soil connectivity for different audiences and produce outcomes that increase the sustainability of soil functions for food production systems. We illustrate our ideas with information from soil-focused agriculture, primarily organic farming and no-till, and knowledge exchange activities.

**Table 1:** The framework of three types of connection between soil and people with examples of the types of connection, the messages provided, the target groups and intended outcomes. The three types of connection are not mutually exclusive

	Type of connection		
	Direct	Indirect	Temporal
Main example of creating connection	Hands-on teaching of visual soil evaluation or description	Information on soil and food production	Information on changes to the environment due to soil degradation
Message	Good soil quality is important for production	Soil is important for food production, food quality and human health	Degraded soil can reduce food security and increase environmental degradation
Target group	Farmers, land managers	Public (e.g. gardeners, cooks, school children)	Policy makers, eco groups
Example intended outcome	Improved soil management	Increase in local food production and of environmental awareness	Adoption of a more sustainable farming system

**Table 2:** Examples of mechanisms of connection to the soil with recommendations for targeted improvements of these within the framework of connections for specific groups. For all groups, drawing parallels between soil functions and personal or community functions increases recognition of the value and vulnerability of the soil resource

Groups	Direct		Indirect		Temporal	
	Mechanism	Improvement	Mechanism	Improvement	Mechanism	Improvement
Farmers/ stakeholders	Working with soil	Field meetings with experts	Soil-focused farming, e.g. organic farming, cover crops, no-till	Social media groups, farmers’ clubs and discussion groups, literature	Long-term agroecological rotations	Long-term monitoring of soil quality and erosion with government education and support
	Visual evaluation	Demonstrations and teaching	Soil health awareness	Field walking and spot soil testing	Better soil management, integrating old and new ideas	Soil-based training, wise use of technology
Policy makers/scientists	Importance of soil in policy documents and research	Meetings with farmer representatives and conferences	Reports on soil science	Social media reporting of research outcomes	Studies of long-term changes	Soil-based training and longer term policy and science funding (5–10 years)
	Visual evaluation/ soil quality	Demonstrations and teaching	Soil quality and ecosystem services	Reports, scientific papers	Long-term soil conservation, agroecological farming	Soil and nutrient status, environmental monitoring initiatives
Society as a whole	Gardens	Community gardens and education	Food and fibre, good soil needed for healthy food	School curricula, social media groups, TV/radio programmes	Ecological and emotional awareness	Eco groups, exhibitions, art work, poetry, soil days/years/decade
	Develop awareness of soil presence	Hands-on training, field walks	Report and demonstrations on soil quality for amenities and gardening	TV and radio programmes, social media showing soil loss, global warming feedbacks through soil	Increased awareness of the long-term importance of soil in society	Soil being incorporated into more environmental exhibitions and popular science programming

## Direct connection

### Visual and tactile examination of soil

Direct connection with the soil is made when we look at it, smell it, handle it or work it. For most, contact with the soil is through gardening or by working with the soil when farming (Table 2). Children in particular like to look for worms and soil arthropods. Smelling the soil connects the assessor to the role of the soil as a holistic, living network of organisms that function in recycling of plant nutrients and production of gas emissions (Shepherd 2009). The appearance of soil is largely determined by its colour and structure, with structure and porosity mainly described from the component parts of the soil or aggregates.

Visual evaluation of soil is a simple test based on description of soil structure where the soil is exposed on a spade or in a profile and pieces are broken up to reveal the soil structure, then described by using a guide and by comparing with reference photographs (Batey et al. 2015). The main methods split between those involving a spade depth of soil, with a focus either on the topsoil, e.g. the Visual Evaluation of Soil Structure (VESS) (Ball et al. 2007) and the Visual Soil Assessment (VSA) (Shepherd 2009) or deeper soil profiles that include both topsoil and subsoil, e.g. ‘Profil Cultural’ (Peigné et al. 2013) and SOILpak (McKenzie 1998). Other, more detailed, soil description systems are exemplified by FAO (2006). A profile method that includes information on soil texture, relief and climate in addition to soil structure into an overall soil quality rating is the Muencheberg Soil Quality Rating System (Mueller et al. 2013). This gives a quality rating between 0 and 100 and is applicable globally.

Soil structure is a key aspect of soil quality that is sensitive to degradation. The small soil blocks used in the VESS and VSA methods are readily portable and make these methods suitable for demonstrating soil structural quality to groups, with both used in agricultural consultancy to increase awareness. The methods integrate information from a spadeful of soil into a single score, a generic indicator of soil quality (Ball et al. 2017) that reveals signs of degradation, such as compaction or other physical damage. Scores are assessed from a key containing photographs and, in the case of VESS, traffic-light grading on a laminated field chart that allows a wide range of users to score their soils quickly. Scores can be used to identify thresholds for soil restoration. VESS has been shown to correlate well with other physical measurements related to soil quality and is one of the ‘core indicators’ of soil quality (Ball et al. 2017) because soil physical conditions determine the habitats of living soil organisms that drive soil processes related to plant nutrient transformations (Beylich et al. 2010). Nevertheless, other properties related to soil biology (e.g. content of earthworms and organic matter) and soil chemistry (e.g. pH and content of plant nutrients and contamination) that provide further direct connection to the soil are required to give a more complete assessment of soil quality and its fertility.

### Application and benefits

Visual evaluation of soil can be used to demonstrate the link between soil quality and crop yield. Mueller et al. (2013) found a linear relationship between cereal grain yield and the Muencheberg Soil Quality Rating at a range of sites where annual nitrogen fertiliser applications were < 100 kg ha^−1^ (close to agroecological systems). The clear inference is that the better the quality of the soil, the better the yield. This is particularly important in agroecological systems such as organic farming where manufactured inorganic fertilisers and pesticides are largely omitted; these require the farmer to have a closer connection with the soil and a metric of soil quality that reflects soil management (Wahlhütter et al. 2016). SRUC had two organic ley-arable rotation trials, at Tulloch near Aberdeen and at Woodside near Elgin in North-east Scotland (Taylor et al. 2006), where there were stocked (mixed) rotations, depending on animals for recycling and grass-clover crops to build fertility. Visual evaluations of soil in 2002, 11 years after the trials began, gave satisfactory scores indicating that structural qualities were unlikely to limit crop productivity. Visual evaluation of soil also allows rapid assessment of spatial variability of soil quality, e.g. in Paraná State, Brazil, no-till soybean yield decreased with VESS soil quality when sampled at intervals along a transect, with the effect being significant at <10% probability level (Giarola et al. 2013). VESS also has benefits of easy comprehension, minimal equipment and ability to be used in remote locations such as the Amazon basin where soil limitations to crop productivity caused by grazing and ‘slash and burn’ management have been identified (Guimarães et al. 2017).

Contact with the soil through gardening can improve health by increased physical activity and, especially in community gardens, by improving social health and community cohesion (Wakefield et al. 2007). Colleagues at SRUC and farmer clients also report that handling and working the soil is a positive and often therapeutic experience that nurtures respect for the soil.

## Indirect soil connection

The second type of connections involves a broader, more intuitive appreciation of the environment with greater awareness of the links between soil and food, ecosystem services, climate change, biodiversity and related decisions (Table 2).

### Health and food

Improving the understanding of the connection between soil and food can be achieved by revealing how the production of healthy food depends on soil and environmental health (Oliver and Gregory 2015). For example, rice is commonly deficient in micronutrients. The content of iron, zinc, copper and manganese in rice grains varied across the three locations of a field experiment in India and were directly related to the soil levels of carbon, copper, zinc, available phosphorus and pH (Pandian et al. 2011).

**Fig. 1:**
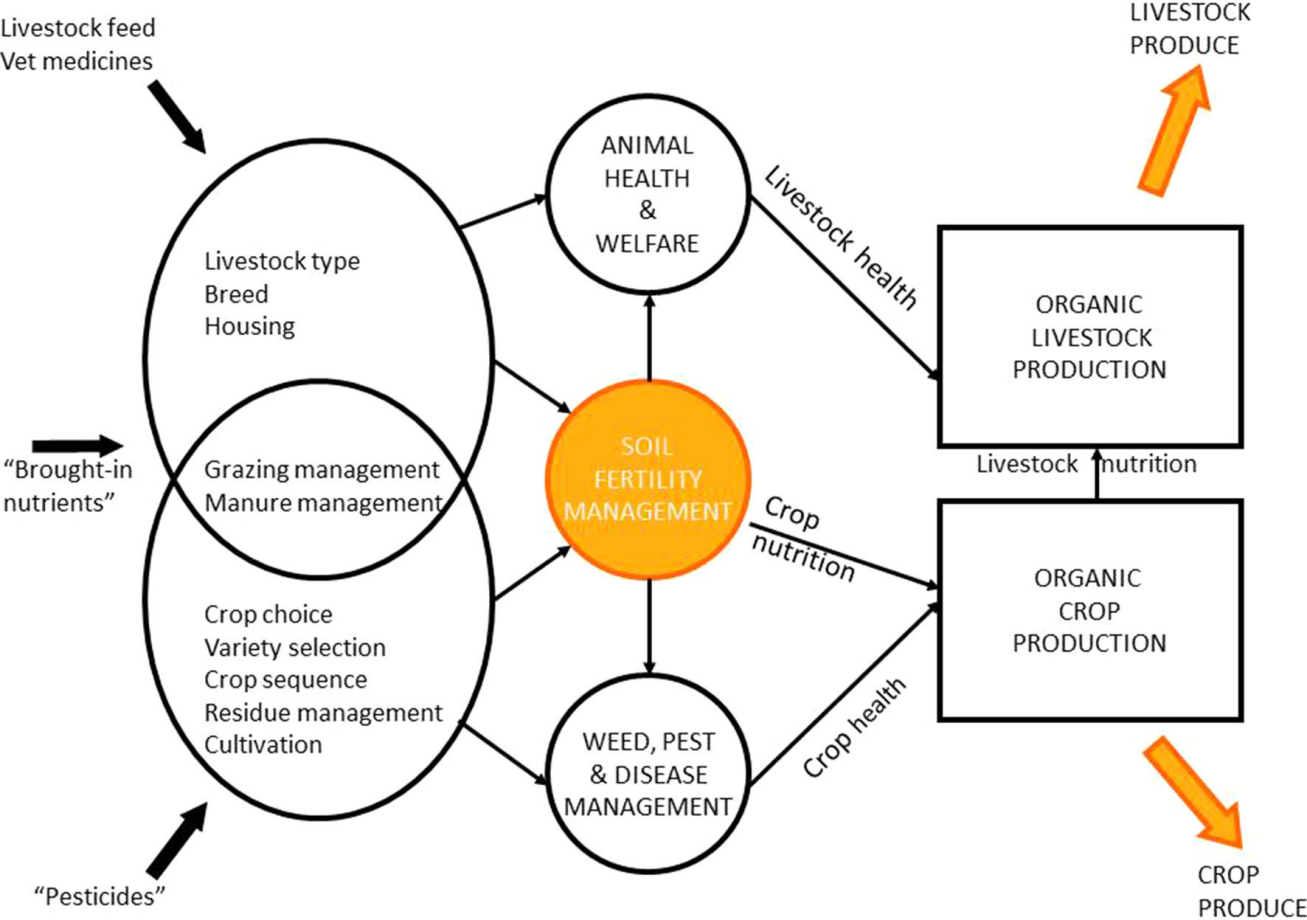
The interactions between soil fertility, farm management, crop and animal produce in organic farming systems. Adapted from a figure in Watson et al. (2002)

A farm can be perceived as an interconnected network linking the nutrition and health of crops, livestock and food quality (Watson et al. 2002). Soil fertility is central to this network (Fig. 1). Reeve et al. (2016) explored the links between soil health, food crop nutritional quality and human health and concluded that organically grown fruit and vegetables contained higher levels of health-promoting phytochemicals but the overlap in management practices among farming systems made generalisations difficult. They stressed the importance of soil management practices (such as organic farming) that enhance soil, plant and human health as important goals for sustainable production systems.

A criticism of organic farming is that yields can be lower than conventional farming in mainstream agronomy (de Ponti et al. 2012). This contrasts with conservation agriculture, in particular no-till, where good yields and yield stability, lower fuel consumption and environmental benefits have led to a rapid global expansion such that 105 million ha were under no-till in 2009 (Derpsch and Friedrich 2009). However, Soane et al. (2012) identified several agronomic constraints in northern Europe associated with soil damage at harvest and the presence of crop residues and weeds. As with organic farming, they concluded that a greater awareness of soil condition is required for the successful application of no-till.

### Ecosystem services

Conservation agriculture practices protect the soil principally by reducing erosion risk, a major factor in South America (Derpsch and Friedrich 2009). Advantages of no-till practised under good conditions (such as where compaction is minimised) include increased aggregate stability, greater organic matter content near the soil surface and improved soil structure and biological activity (Soane et al. 2012). Stavi et al. (2016) produced agro-environmental scores based on ecosystem services and soil functions for different farming systems, and these were highest for conservation systems. Ecosystem services provided by the soil are thus important indirect connections to the soil.

### Application and benefits

Commitment to agroecological systems can be worthwhile for direct food production. In the SRUC organic rotations trial at Tulloch, where a stocked (mixed) rotation gave economically sustainable crops of oats (Table 3) and grass/clover, a stockless rotation was added in 2007 to produce further crops either for direct human consumption or those that could be fed to animals. Contrary to expectations for a soil of only moderate fertility (Taylor et al. 2006), and possibly due to the dedication of the soil scientists running the trial, the stockless rotation has produced yields of beans, potatoes and barley over 8 years’ continuous operation (Table 3) that are good or moderate for the soil and climatic conditions (Lampkin et al. 2006). Nevertheless, specific problems of disease, predation by birds and invertebrates and extremely wet weather could decrease yield.

**Table 3:** Average yields of oats in a stocked rotation and of other food crops in a stockless organic rotation in North-east Scotland (unpublished data). The beans failed to grow sufficiently to provide harvestable yields in 2007–2009 and 2012 because of disease and invertebrate predation. DM = dry matter

	Stocked rotation	Stockless rotation		
	Oats (t ha^−1^ grain @85% DM)	Beans (t ha^−1^ grain @85% DM)	Potatoes (t ha^−1^ fresh weight)	Wheat (t ha^−1^ grain @85% DM)
Average yield over 2007–2015	4.0	3.0	15.2	2.7
Standard deviation over 2007–2015	1.7	2.3	8.4	1.0

Agroecological systems can also improve most ecosystem services and functions of the soil (Halberg et al. 2015; Lampkin et al. 2015). In the long-term SRUC trial at Tulloch, average soil carbon content across all plots increased from 40.3 (±1.9) to 50 (±1.6) g kg^−1^ over the first 15 years of the experiment, with the emissions of greenhouse gases in the experiment generally less than in comparable conventional systems, particularly in grassland (Ball et al. 2002). However, timing of management interventions such as ploughing out of grass-leys and manure applications was critical to the success of the organic system during weather extremes associated with climate change (Ball et al. 2014).

### Holism and networking

Ecology, or more specifically deep ecology as defined by Naess (1973), realises the world as networks of phenomena that are fundamentally connected and interdependent. Grunwald et al. (2017) proposed that growing ecological awareness is necessary to value, care for and secure the natural world including soils. This growth acts through individual and collective interior values such as beliefs, values, cultures and spirituality. Here we propose the idea that the connection of humankind with soil is facilitated by the use of soil as a metaphor of networking because within soil all creatures fulfil roles and niches that are complementary in a functioning ecosystem. The parallel in both a farm and in society is a circular economy underpinned by agroecological approaches that restrict consumption and waste within ecological limits.

**Fig. 2:**
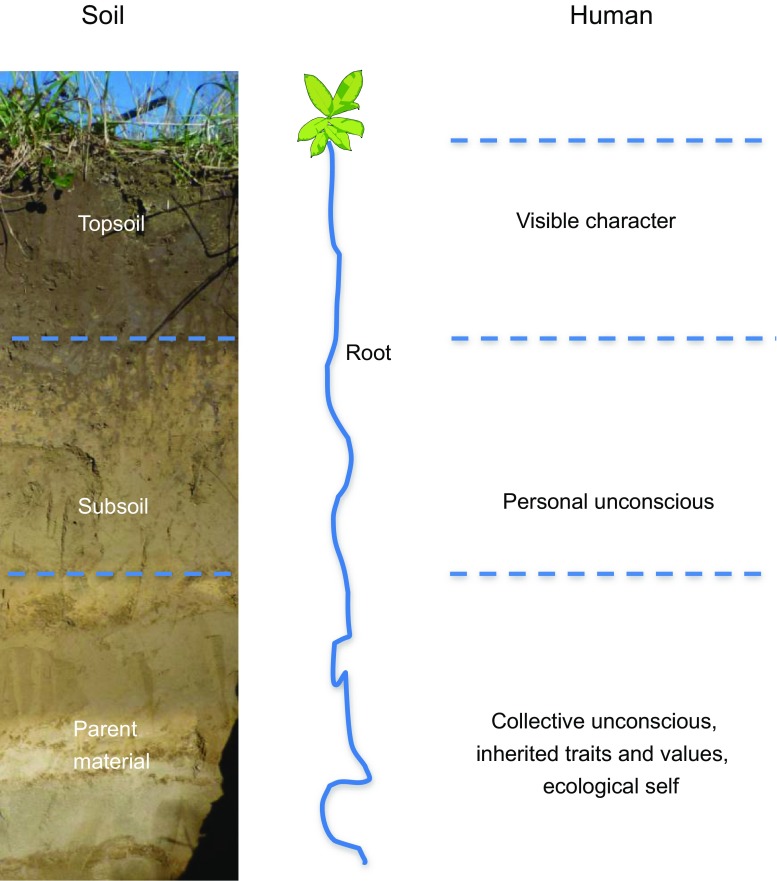
A holistic model of the human psyche based on soil. The horizons of a typical soil profile (left) resemble the different levels of expression of the human self (right). Adapted from a figure in Ball (2015)

Parallels and connections between the functions of soil and those of the human body were identified by Patzel (2010) and by Hans Jenny as elaborated by Logan (1995). We extend this approach to using the soil profile as an holistic model of the structure of the human psyche. This is used to show how decision making can go beyond the superficial demands of our egos by restoration of the connection to what Naess (1973) calls the deep or ecological self or which Grunwald et al. (2017) call interior individual and collective perspectives which can stimulate environmentally responsible behaviour. These resemble the endogenous personal traits and values considered by Olver and Mooradian (2003) to bring an openness to change. The three horizons of the profile represent levels of consciousness based on Jungian concepts (Fig. 2). These are similar to the three types of self-identified by McIntosh (2008) as the conscious self, the shadow self and the deep self. In this way, we propose a novel connection to the soil at a profound level.

At the surface, the topsoil represents the visible character, ego or consciousness of the person. The second horizon or subsoil is similar to the personal unconscious where hidden potential is stored; it underlies our conscious lives and contains our emotional inner images and ideas which influence our actions unconsciously (Patzel 2010). The bottom or third horizon (parent material) is the collective unconscious that contains hidden traits and core values inherited from parents and ancestors. It also represents the ecological self and can be seen as a continuum linking us with others through networks. Further details on the parallels between soil and humankind are given in Ball (2015).

### Application and benefits

Novel approaches to telling the story of the soil that include the anthropomorphism and philosophy developed here such as by Logan (1995), Patzel (2010) or Ball (2015), help to nurture indirect connection. Awareness of the value of the soil as a resource can bring a deeper perception in land users connected to shared inherited values (Fig. 2) (Wahlhütter et al. 2016) and this can help expand and remould the knowledge that farmers already possess (Shaxson 2006). Perceiving workings of the soil similar to those of the mind should bring a closer appreciation of the importance of what is happening below the soil surface and reinforce to farmers the relevance of the nature of the subsoil and how it supplies basic nutrients to sustain the topsoil and needs to be cared for in order to conserve topsoil functions.

Fostering deeper connections with the soil through the Jungian model (Fig. 2) increases the chances of developing affective connection (empathy and emotional affinity) with the natural environment that can bring about pro-environmental behaviour (Hinds and Sparks 2008). When this is shared with others in groups it can help to make choices that conserve the environment for future use, whilst sustaining yield. Nurturing the desire to move towards more soil-focused farming may result from improved connectivity with the soil; awareness of its condition and potential for improvement can increase the emphasis of land management to care and safeguard, the main principles of soil husbandry (Batey 1988). In our model of the human psyche (Fig. 2), the process of soil restoration involves deepening of the topsoil layer or increasing root growth and has a parallel in developing personal awareness and community spirit by restoring contact with our own endogenous traits (Olver and Mooradian 2003).

## Temporal connection

Temporal connections bring awareness of the dynamics of soil processes and soil management (Fig. 1; Table 2). It can commonly be perceived from changes in soil fertility as a soil ages. Soils in India, Asia and other tropical countries are older than those in Northern Europe and America so that they have lower baseline fertility. Intensive cropping of older soils can cause a relatively rapid loss of fertility as nutrients are removed from the system without being replaced (Jones et al. 2013).

Temporal soil connection is also revealed by physical changes in soil quality that are linked to crop productivity and ecosystem services in response to soil management and how these are likely to continue into the future. Under no-tillage in south-east Scotland in an SAC long-term experiment, grain yields varied from year to year due to the interaction of weather and soil type, but showed no overall time trend under no-till or under conventional tillage (Soane and Ball 1998). However, soil organic matter at 0–60 mm depth under no-till increased from 37 to 49 g kg^−1^ between 1975 and 1990 in the half of the experiment under a gleysol, but remained at 50 g kg^−1^ during the same period under a Cambisol.

**Fig. 3:**
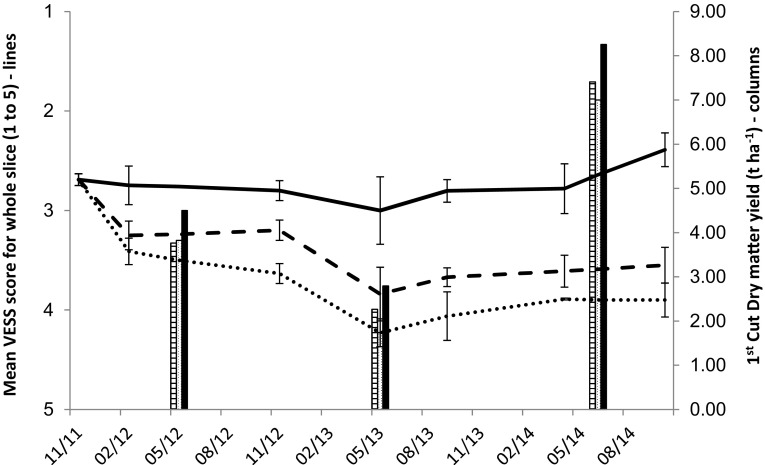
The change in VESS structure scores (1 is best, 5 is worst) and grass silage dry matter yields (t ha^−1^) from November 2011 through to September 2014, with annual application of compaction treatments by tractor (dotted line and middle column), by trampling with dairy heifers (dashed line and left hand column) and no compaction (solid line and right hand column). The ground pressure of both heifers and tractor was 200–250 kPa. The bars represent standard error of the mean. The VESS scores were first published in Ball et al. (2017). Yield data are unpublished

VESS assessments in an SRUC compaction experiment based on a grassland sward located in south-west Scotland showed a decrease in quality over time due to compaction. The no compaction treatment corresponded to soil-focused farming, in that compaction by animals and tractors was minimised, to diminish soil structural degradation over the 3 years of the experiment (Fig. 3). Although the grass yields varied between years, mainly reflecting spring weather conditions before the first silage cut, the no compaction soil always yielded significantly greater plant dry matter than soils either trampled by heifers or compacted by a tractor. Reduced compaction also resulted in a significantly lower peak nitrous oxide flux in March 2012 (7.8 g N ha^−1^ day^−1^) than for the trampled soils (*P* < 0.01, 18.6 g N ha^−1^ day^−1^) and the tractor compacted soils (*P* < 0.001, 25.0 g N ha^−1^ day^−1^) (Ball et al. 2013). This effect was partly attributed to the compacted soil retaining more water in the pores giving more anaerobic conditions and greater nitrous oxide emissions than the non-compacted soil. Similarly, at Tulloch in the ley-arable rotation under mixed cropping, soil structural quality improved during the fertility-building period (3–4 years) under grass-clover and decreased with tillage during the fertility depletion period (2–3 years) under arable cropping (Ball and Douglas 2003).

Temporal connection to soil is also shown by the influence of climate change that increases susceptibility to soil degradation in response to increases in mean annual temperature and decreases in precipitation (Lal 2009b). A temporal soil connection also exists in the minds of farmers and workers of the land, who provide the link between past and future management. Farmer experience and knowledge of the land that is accumulated, tested and deeply embedded in indigenous cultures and traditions requires nurturing and support to help safeguard this for the future (Tenywa et al. 2013). On the Outer Hebrides of Scotland, the restoration of neglected organic soils to agricultural production is restricted by the loss of the crofters over the past 50–70 years who knew how to manage the land. Visual methods to examine the soil, vegetation and the landscape can be used to provide clues for restoration both of the land and of the crofting community (Scherbatskoy et al. 2015).

Traditional indigenous knowledge can be adapted to permit the wise use of modern and innovative techniques (Lal 2009c), though this may prove difficult for inexperienced or poorly educated farmers and lost to those in training and waiting to get on to the farming ladder.

### Applications and benefits

Temporal connections are likely to be especially important in agroecological systems and no-till systems where they help in developing long-term soil resilience (de Moraes Sa et al. 2013; Tenywa et al. 2013; Nezomba et al. 2015). Such systems also major on the recycling and recovery of nutrients that help mitigate the progressive loss of micronutrients on older soils (Jones et al. 2013). Temporal connection to soil by regular monitoring of soil quality would preserve the long-term sustainability of soil function and maintain the security of the soil. Thus long-term knowledge of the influence of agroecological systems on soil quality and crop yield and quality is important and shows the importance of long-term field experiments such as the SRUC organic rotations trial near Aberdeen (Taylor et al. 2006), the DOK trial in Switzerland (Flieβbach et al. 2007) and Broadbalk at Rothamsted Research, UK (Watts et al. 2006).

## Discussion

### The importance of a framework approach to soil connection

The framework structure of connections (Table 1) provides a ready means of understanding the role of soil connection in building awareness of the soil as an essential resource within a network linking agriculture, people and the environment. It shows, at a glance, the importance of transforming agriculture by sensible soil management for food security, prevention of soil degradation and soil restoration. The success of the SRUC rotational and tillage experiments was dependent on a high standard of management (Ball et al. 2014), a common theme in other experiments. Snapp et al. (2010) found that management intensity was more important than crop diversity for the success of yield and carbon sequestration in a long-term organic experiment in Michigan. Martini et al. (2004) also reported that yield increased during the first 3 years of transition to organic farming as a result of improved experience and management, rather than better soil quality.

Our framework for the development of a soil-based perception for better soil management to sustain the food system may be achieved not only through direct soil quality and management effects but also through the use of the soil to connect to value systems [e.g. the ideas deep within us and soil–people analogies (Fig. 2)] and underpins the mechanisms of connection to the soil (Table 2). This area is considered by Wahlhütter et al. (2016) to require further development such that shared identities or strategies for ‘good’ farming and nutrition can be cultivated. Hinds and Sparks (2008) also found that such affective connections to the environment were significant predictors of intentions to engage with the natural environment. In applying similar ideas to food production, Tudge (2016) stressed the importance of developing ‘enlightened agriculture’ where a change in mind-set comes about using concepts of intuition, collaboration, trust and coherence as important drivers of the food system. Such approaches could foster the development of bottom-up, farmer- and consumer-led initiatives called for in IPES-Food (2016).

The framework shows the importance and time dependence of soil management emphasising good quality soil in sustaining agriculture and soil functions in relation to ecosystem services, particularly with agroecology. These are critical to improving the food production system (Jones et al. 2013) where the manipulation of nutrient availability and conservation of nutrient stocks are vital (Watson et al. 2012), especially for the restoration of resilience in degraded soils (Lal 2009b).

The different types of connection are not mutually exclusive and do interact. For example quantifying temporal connections may require the use of direct connections of visual evaluation of soil. Also mechanisms for delivery of information on soil often use a mixture of types of soil connection. Nevertheless the distinction between the three types of soil connection strengthens the awareness of the importance of soil in different areas of activity.

### Promoting connections to the soil

We summarise in Table 2 our recommendations for improvement of mechanisms of connection to the soil within the framework of connections (Table 1) for the three groups of people identified. Specific outcomes for some of these methods of connection are shown in Table 4.

**Table 4:** Outcomes of selected methods of improved connection to the soil for different groups

Groups	Method	Outcome	Examples
Farmers/stakeholders	Field training day; talk plus visual evaluation training (direct)	Stimulates further assessments (e.g. nutrient budgeting) and further guidance for planned changes (e.g. compaction control, cover crops, farming by visual evaluation)	Field day on maximising soil and meeting land management rules http://www.nzherald.co.nz/the-country/news/article.cfm?c_id=16&objectid=11854007 Soil compaction: problems and remedies https://www.youtube.com/watch?v=Xgwr9yAJ2XA McKenzie (2013) Visual soil examination for farm evaluation Shepherd (2009) Visual Soil Assessment
Policy makers/scientists	Training day: talk plus sampling/visual evaluation training (direct/temporal)	Increases soil aspects of environmental monitoring (e.g. water quality); soils included as a multidisciplinary component of research	Regulation of diffuse pollution http://www.sepa.org.uk/regulations/water/diffuse-pollution/ Soil Functions https://www.ceh.ac.uk/our-science/science-areas/soil
Society as a whole: adults (1)	Organic farm/community garden walk or workshop with demonstration of soil (indirect)	Stimulates interest in gardening and local produce	Transforming the way we farm https://www.soilassociation.org/our-campaigns/better-food/transforming-the-way-we-farm/
Society as a whole: adults (2)	Talk or exhibition on soil that encompasses food, farming, art, behaviour change (indirect)	Greater awareness of the soil as an important entity with unifying attributes	Soil Saturdays https://vimeo.com/147749985 Soil Culture http://www.exeter.ac.uk/esi/research/creativeexchangeprogramme/soilculture/ Soil Culture: bringing the arts down to Earth http://www.cornerhousepublications.org/publications/soil-culture-bringing-the-arts-down-to-earth/
Society as a whole: children (1)	Interactive exhibits showing soil creatures and anthropomorphic soil types (indirect)	Stimulates environmental awareness and living links to food	DIG IT The Secrets of Soil https://www.soils.org/discover-soils/dig-it
Society as a whole: children (2)	Environmental award schemes and badges related to soil functions (indirect)	Improved understanding of why soils are important in global issues	FAO Soils Challenge badge used by Scouts http://www.fao.org/3/a-i3855e.pdf

#### Farmers and stakeholders

Farmers and stakeholders benefit from the improved direct connections of working with the soil and visually assessing it in the field (Table 2). In our experience as experts working with groups of farmers on field training days where they handle soils, this not only increases awareness of its value as an asset but also stimulates the development and sharing of ideas and experiences. This can prompt them to plan further soil-based improvements through measurements (Table 4) and to share ideas for innovation in agriculture (Ball et al. 2017). Field training days have involved scientists working with farmers and consultants via the Scottish Agricultural College (now Scotland’s Rural College), the Danish Farm Advisory Service, Soil Association Climate Change Programme, England Catchment Sensitive Farming Delivery Initiative, supermarkets and the Farming for a Better Climate Initiative.

Indirect connection to the soil provided by information on soil-friendly agriculture through literature, discussion groups, social media apps (Table 2) can lead to soil management changes ranging from adopting cover crops to application of complete agronomic management systems based on visual soil and crop evaluation (Shepherd 2009). Farming discussion groups are an important mechanism for communicating the efforts of high-profile individual farmers such as Allan Savory and Joel Salatin who promote mixed farming systems for conservation of soil carbon and fertility (Ohlson 2014). Connection through good knowledge exchange between large- and small-scale farmers is also important, for example, a Canadian experience with no-tillage helped soil conservation with small farms in China (Lafond et al. 2009). Revitalising the temporal connection between the elders and a younger generation (McIntosh 2008) may help to carry over wisdom and to place inherited knowledge in a modern context, allowing for successful adaptation of novel conservation agriculture techniques (Lal 2009c). The need for improved soil connection for better soil management (Table 2) can also be tackled, for potential soil users, by the co-ordination of education and extension systems to provide training in soil management for a new generation of soil specialists as identified by FAO and ITPS (2015). These would be ‘knowledge brokers’ trained in soil management that overcome soil-related barriers to productivity and soil function (McKenzie et al. 2015). This would enable closer collaboration between farmers and researchers in developing research initiatives and spreading agroecological knowledge (Lampkin et al. 2015).

#### Policy makers and scientists

Policy makers and scientists benefit from teaching and demonstrations about soil by visual evaluation and from laboratory assessments of soil quality (direct connection), by reading reports and scientific papers (indirect connection) (Table 2) and targeted improvements in environmental monitoring (Table 4). This can lead to greater inclusion of soil aspects in agricultural and land environmental research (e.g. nitrate leaching, Vibart et al. (2016)) and to long-term monitoring of soil quality and related environmental aspects (Table 4).

The importance of soil nutrient recycling within networks for sustainable food production highlights the relevance of life-cycle analysis for assessing sustainability in agricultural systems (Nemecek 2011). However, nutrient mining, especially of micronutrients, will continue due to increasing urbanisation of populations, making it difficult to transport and recycle the nutrients from human wastes. A wider, more radical ‘whole systems’ approach, based on nutrient stocks and their conservation (Jones et al. 2013), is likely to be needed to re-design current food production systems.

We have shown that a direct connection to the soil by visual evaluation can help to identify land areas requiring improved soil management (McKenzie 2013). This clearly has the potential to contribute to the improved observation systems required to monitor progress in the development of soil awareness, education and of management systems called for by FAO and ITPS (2015) to ensure soil security.

#### Society as a whole

The public who are gardeners and who are curious about the soil can develop direct connections by, for example, learning ‘hands-on’ within community gardens and taking guided country or farm walks. Such approaches may explain why these methods have had a significant impact not only on farmers, but also on gardeners, the general public, students and schoolchildren (DeLind 2002; Rojas et al. 2011; REF 2014; Brevik et al. 2015).

This awareness can lead to understanding the benefits of agroecology and ecosystem services that in turn bring social benefits (Gregory et al. 2016).

**Fig. 4:**
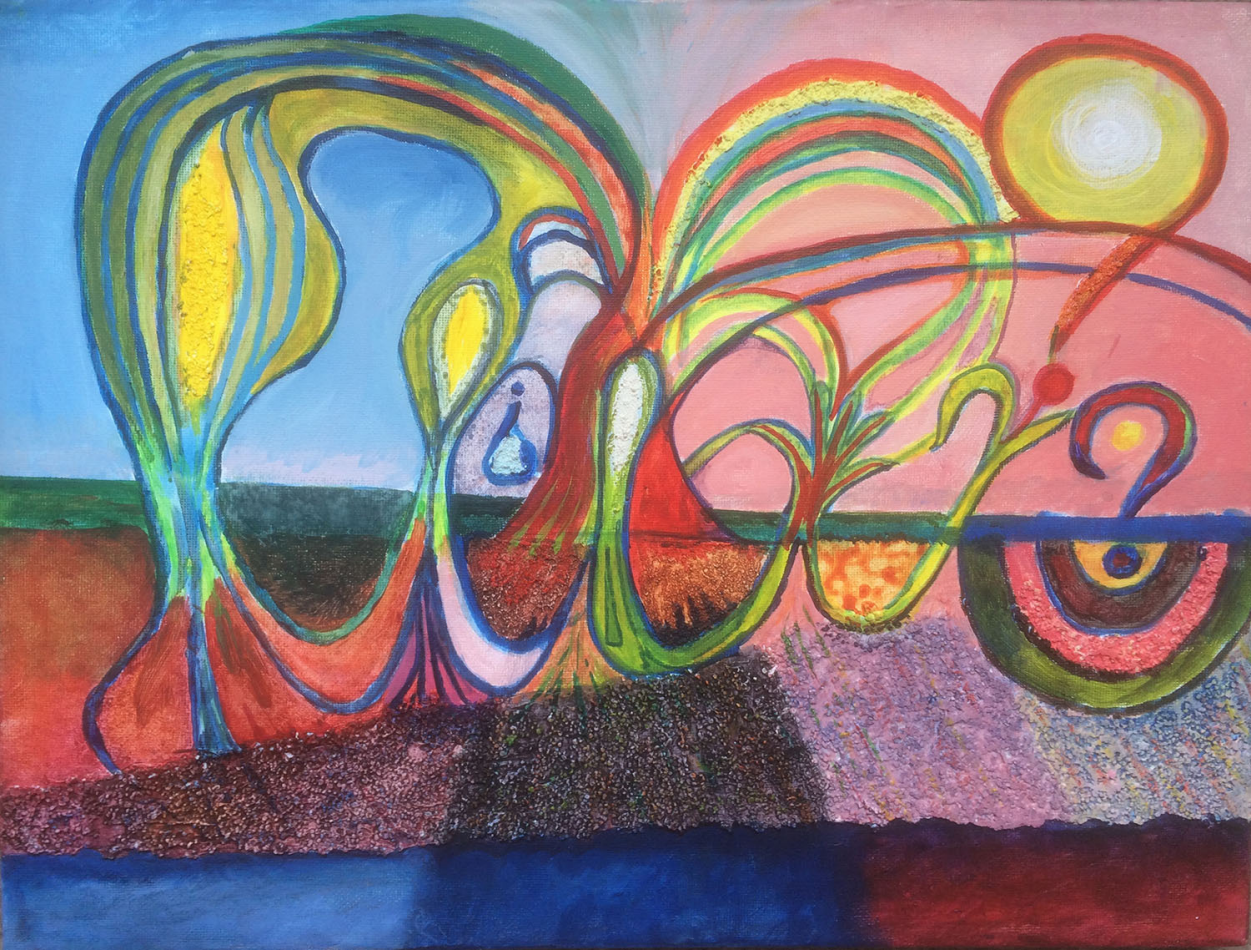
Soil depletion. Acrylic on canvas by Bruce Ball and Tom Henry. Temporal connection to the soil is shown as a sideways view of a soil profile as it is progressively depleted (left to right) by loss of nutrients as gases and leaching, loss of stored water and thinning of the topsoil due to global change and agricultural activity

For the public in general, where there is a perceived detachment between food and its source, particularly for large urban communities (Kearney 2010), increasing awareness of soil is likely to be through the development of indirect connections. These include improvement in soil education that includes the removal of long-held meanings associated with soil as dirty or tarnished (Harrison et al. 2012). This can occur through promoting links between soil and healthy plants, food and drink and the links between soil and global change (Acton and Gregorich 1995; Lal 2004). Community gardens can help to develop the connection with the soil because of the importance of soil and nutrient maintenance and the role of agroecology for their success (Gregory et al. 2016). These can be improved by programmes in the media and social media groups (Table 4) that stimulate interest in gardening and in local produce (Williams and Brown 2012). Indirect connections such as talks, poetry or art work that shows the importance or vulnerability of soil (Fig. 4) and how it can have unifying and networking attributes relevant to behaviour (Fig. 2) should engage the emotions and show the importance of soil as a living entity (Tables 2, 4). Soil workshops and talks, given by the authors, that stress the importance of both direct and indirect connections to the soil have been attended by groups of students, artists, eco-church groups and environmental organisations. Examples include the Scottish Sculpture Workshop on ‘Petro-Subjectivity’, Lumsden, UK; the Guild of the Church of Scotland, Midlothian, UK; the Organic Research Centre, Bristol, UK and MSc students at ISARA, Lyon, France. Many participants reported that these events provided inspiration on the relevance of soil both to food production and to life in general.

Temporal connections with the soil can also help the public to realise the influences of climate change and agriculture on soil degradation. Awareness through art and cultural initiatives can further reveal the vital, ecological importance of soil (CCANW 2016). Art can help to show the vulnerability of soil to degradation as the soil thins and becomes depleted of nutrients and moisture by erosion, leaching and greenhouse gas emissions (Fig. 4). In the longer term, such improved connections will help bring about the recognised need for a change in mind-sets of people to achieve the increasingly unavoidable goals of moving to a low carbon economy and reducing consumption of resources.

Fostering connection to soil needs to begin with childhood. The inclusion of soil in school curricula is important as are initiatives by volunteer organisations that lead to improved understanding of the importance of soil to food and environmental conservation (Table 4). At a recent meeting at an organic farming centre in South Scotland, as part of the ‘Global Food Citizens’ initiative, primary school teachers identified these words associated with soil: ‘essential’, ‘minerals’, ‘worms’, ‘peaty’, ‘mess’, ‘a workforce’, ‘vital’, ‘a mystery’, ‘alive’, ‘forgiving’ and ‘connection’.

## Conclusion

Examples of knowledge exchange and research efforts on soils are put into the context of connections between the soil and different groups of people. Allocation of connections to the soil into three types and identification of their relevance to different groups of people allow improved recognition of the different roles of soil and soil management to the food system and environmental conservation. The needed improvements of these connections have been identified and recommendations range from field training to behaviour change. Likely outcomes encompass further connections through ‘soil-based farming’, the stimulation of garden food production to recognition of soil as a unifying entity.
